# Effect of Starch Loading on the Thermo-Mechanical and Morphological Properties of Polyurethane Composites

**DOI:** 10.3390/ma10070777

**Published:** 2017-07-10

**Authors:** Tayser Sumer Gaaz, Abu Bakar Sulong, M. N. M. Ansari, Abdul Amir H. Kadhum, Ahmed A. Al-Amiery, Mohamed H. Nassir

**Affiliations:** 1 Department of Mechanical & Materials Engineering, Faculty of Engineering & Built Environment, University Kebangsaan Malaysia, Bangi 43600, Selangor, Malaysia; 2 Department of Machinery Equipment Engineering Techniques, Technical College Al-Musaib, Al-Furat Al-Awsat Technical University, Al-Musaib, Babil 51009, Iraq; 3 Center for Advanced Materials, College of Engineering, Universiti Tenaga Nasional, Kajang 43000, Selangor, Malaysia; ansari@uniten.edu.my; 4 Department of Chemical & Process Engineering, Faculty of Engineering & Built Environment, Universiti Kebangsaan Malaysia, Bangi 43600, Selangor, Malaysia; amir8@ukm.edu.my; 5 Energy and Renewable Energies Technology Center, University of Technology, Baghdad 10001, Iraq; dr.ahmed1975@gmail.com; 6 Program of Chemical Engineering, Taylor’s University-Lakeside Campus, Subang Jaya 47500, Selangor, Malaysia; mohamedh.nassir@taylors.edu.my

**Keywords:** starch, composite, thermal stability, mechanical properties, polyurethane

## Abstract

The advancements in material science and technology have made polyurethane (PU) one of the most important renewable polymers. Enhancing the physio-chemical and mechanical properties of PU has become the theme of this and many other studies. One of these enhancements was carried out by adding starch to PU to form new renewable materials called polyurethane-starch composites (PUS). In this study, PUS composites are prepared by adding starch at 0.5, 1.0, 1.5, and 2.0 wt.% to a PU matrix. The mechanical, thermal, and morphological properties of PU and PUS composites were investigated. Scanning electron microscope (SEM) images of PU and PUS fractured surfaces show cracks and agglomeration in PUS at 1.5 wt.% starch. The thermo-mechanical properties of the PUS composites were improved as starch content increased to 1.5 wt.% and declined by more starch loading. Despite this reduction, the mechanical properties were still better than that of neat PU. The mechanical strength increased as starch content increased to 1.5 wt.%. The tensile, flexural, and impact strengths of the PUS composites were found to be 9.62 MPa, 126.04 MPa, and 12.87 × 10^−3^ J/mm^2^, respectively, at 1.5 wt.% starch. Thermal studies showed that the thermal stability and crystallization temperature of the PUS composites increased compared to that of PU. The loss modulus curves showed that neat PU crystallizes at 124 °C and at 127 °C for PUS-0.5 wt.% and rises with increasing loading from 0.5 to 2 wt.%.

## 1. Introduction

Starch (amylum) is a natural renewable polysaccharide polymer obtained from a great variety of crops and is a promising raw material for producing biodegradable products [1,2,3]. Starch is a polymeric carbohydrate of a large number of glucose units joined together by glycoside bonds and is commonly found inhuman diets in foods such as potatoes, wheat, corn, and rice [4,5]. Chemically, starch consists of linear and helical amylose and branched amylopectin. The availability and the low cost of starch made it a strong competitor to petroleum based thermoplastics in many applications [6,7]. Starch is currently used as filler with a synthetic polymer in the field of thermoplastic starch (TPS) to enhance the mechanical properties by improving the crystallinity of the constituting polymers [4].

Polyurethanes (PUs or PURs) are used as host polymers for many applications. PU is composed of organic units called urethane and appears in two types: thermoset and thermoplastic polymer [8,9]. PUs are formed as a result of chemical reaction of a di-(isocyanate) and polyol. A new type of PUs, called non-isocyanate based polyurethane (NIPUs) has been synthesized by avoiding using isocyanates—the toxic material [1]; however, it has been rarely found in applications. In another important application, the functional material thermo-sensitive PUs were found to have the ability to sense and respond to external thermo-stimuli in a predetermined temperature range [10].

During the past 40 years, there have been numerous studies and excellent publications on the preparation and the structural, thermal, mechanical, and morphological characterization of TPU systems by various research groups [11,12]. PUs are one of the important classes of polymeric materials that have various applications such as biomedical, construction, textile, automotive, insulating materials, adhesives, and commercial molded components [1,13,14,15]. The potential impact of PUs continues to be strong and promising in many emerging fields such as biomaterials, tissue engineering, optoelectronics, shape-memory materials, conducting polymers, molecular recognition, and smart surfaces [16,17]. In particular, the ability of PU as a support to promote new tissue formation in excisional wounds in rat skin based on platelet-derived growth factor (PDGF) has shown a potential utility of biodegradable PUs both as a supportive and as a protein delivery system for tissue restoration [18]. Generally, the remarkable applications of PU in the medical field are due to simple fabrication, biostability, and strong electrical insulation. However, the remarkable applications are restrained by PU degrading, which results in deep cracking, stiffening, erosion, or the deterioration of mechanical properties such as tensile and flexural strength. Despite these drawbacks, the future of PU composites and nanocomposites is very optimistic in producing soft and tough biomedical implants [19,20,21]. Starch reaction with the low glass temperature polymers produces amorphous elastomer networks which swell in dry dimethylsulfoxide (DMSO) and tetrahydrofuran (THF) and possess a reduced hydrophilicity [22]. These materials could be used as matrices for composites with very good mechanical properties without the need of surface treatment [1]. Recently, shape applications have been progressing for designing biomedical implant devices due to their thermal response behavior [23]. The modified TPS are currently under investigation using a solid-state reaction combined with intensive mixing conditions [23]. Hetflejš et al. [24] found that at room temperature the protection was very slow and required several days for completion. It has been seen that the stabilizers act simultaneously as molecular weight regulators. It was found that the DSC or TGA onset temperatures of oxidation increased with increasing molar concentration of the stabilizers [24]. The mechanical properties of the available thermoplastic starch films are still far behind meeting the appropriate surface hydrophobicity, water vapor barrier, and adequate mechanical properties [23].

In this report, PU and PUS composites at different percentages of starch additive were prepared and investigated. The investigation included mechanical properties, physical appearance and morphology, storage capacity, and thermal properties. The study adds very important results regarding the mechanical properties which could enhance the applications of these new composites.

## 2. Results and Discussion

### 2.1. SEM of Starch and Neat PU

**Figure 1:**
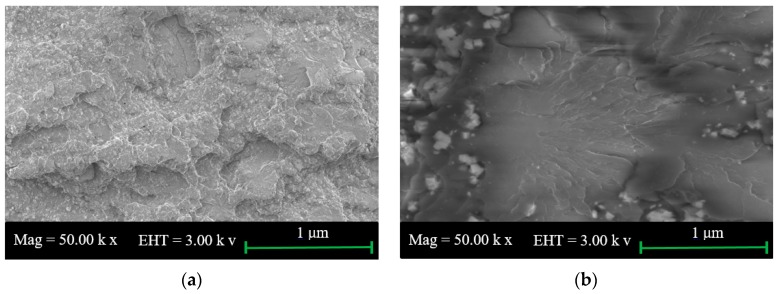
Scanning electron microscopy (SEM) microphotographs for (**a**) polyurethane (PU) and (**b**) starch.

The morphologies of the PU surface and starch powder are shown in Figure 1a,b, respectively. The neat PU sample was prepared by dropping a small mass of PU on a cleaned glass surface while the starch powder was spread over another glass surface. Both surfaces are smooth and do not show noticeable irregularities, in agreement with other previous studies [1,22].

### 2.2. SEM for Tensile and Impact Fractured Surface of PUS

**Figure 2:**
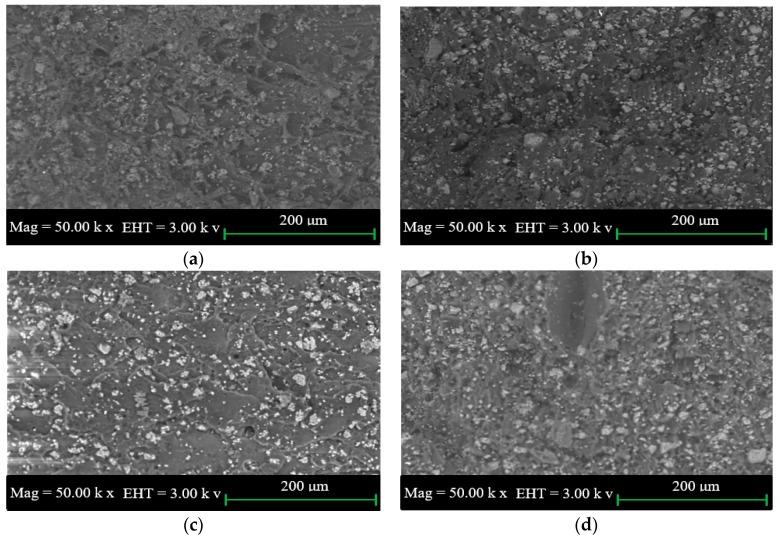
SEM images of the tensile fracture of the polyurethane-starch (PUS) composite at starch wt.% of (**a**) 0.5; (**b**) 1.0; (**c**) 1.5; and (**d**) 2.0.

The SEM images of the tensile fractured surface of PUS composites at different starch loadings are shown in Figure 2a–d. The fractured surfaces show the presence of starch which is represented by white dots distributed over the PU surface. As the starch percentage loading increases, the density of the white dots and, to a lesser extent the size, increase. The increase in the size suggests the formation of agglomeration which has a significant effect on the physio-mechanical properties of the composites. The fractured tensile surface at 0.5 wt.% starch (Figure 2a) does not show clear cracks in the fractured surface. The crack in the surface becomes very clear as the starch loading increases from 1.0 to 2.0 wt.% starch as shown in Figure 2b–d. SEM images shown in Figure 2 suggest that the tensile strength increases and then decreases due to the formation of the cracks which confirms the dependency between the tensile strength and the agglomeration of starch in PU matrix.

**Figure 3:**
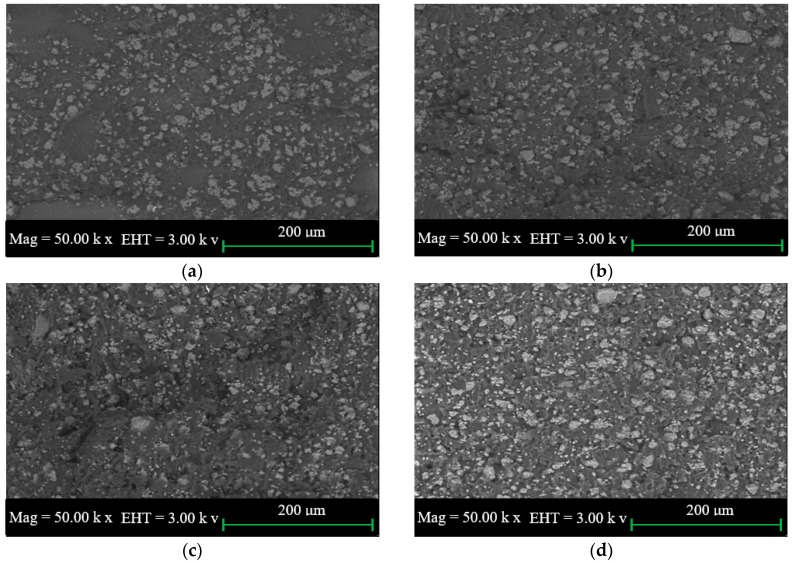
SEM images of impact fracture of PUS for starch wt.% of (**a**) 0.5; (**b**) 1.0; (**c**) 1.5; and (**d**) 2.0.

The SEM images of the impact fractured surfaces of PUS composites at different starch loadings are shown in Figure 3a–d. The similarity of the SEM images of the impact fractured surface and the tensile fractured surface is characterized by the distribution of starch over the PU surface and the presence of agglomerated starch. However, SEM images of the impact fractured surfaces do not show the presence of cracks, possibly because of the sudden cut of the surface rather than slow cutting in case of the tensile fracture. SEM images, again, support that the mechanical properties improve until the onset of the agglomeration on PU-starch matrix.

### 2.3. Tensile Test

**Figure 4:**
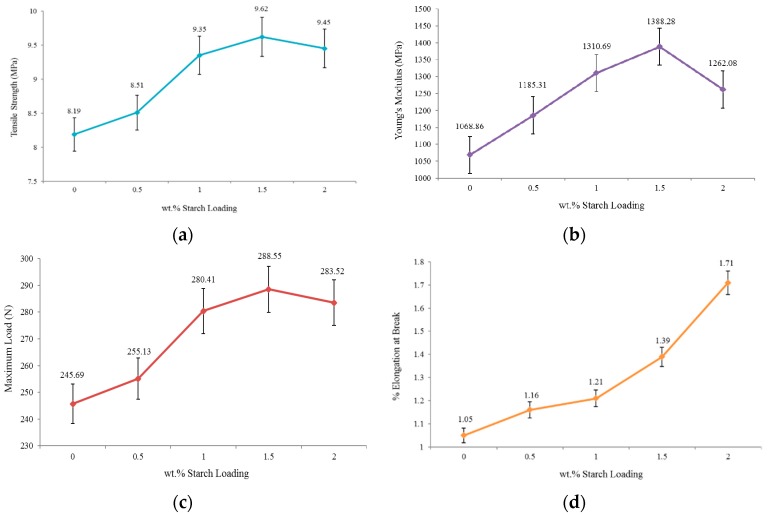
Mechanical tests of PU and PUS (**a**) tensile strength; (**b**) Young’s modulus; (**c**) maximum load; and (**d**) elongation at break.

The results of the tensile strength of neat PU, PUS-0.5%, PUS-1.0%, PUS-1.5%, and PUS-2.0% are shown in Figure 4a. The thermostat PU exhibits a tensile strength of 8.19 MPa. As the starch filler increases from 0.5 to 1.5 wt.%, the tensile strength increases, attaining the maximum value of 11.62 MPa, showing a significant percentage increase of about 17%. Then, the tensile strength decreases as the starch amount increases to 2.0 wt.% at which point the tensile strength declined to 9.45 MPa. The behavior of PUS composites could be understood based on the versatile behavior of PU which is susceptible to change due to external parameters such as temperature, and recently, by adding small quantities of foreign materials. PU attains its highest rigidity at a 1.5 wt.% starch content, and again it loses its rigidity and becomes plastic/rubber-like as the starch percentage increases [25]. Based on the SEM images shown in Figure 3, the formation of the agglomerated starch adversely affected the improvement of the mechanical properties. This behavior of attaining maximum tensile strength followed by a reduction at certain starch loading percentages, combined with the formation of starch agglomeration as seen in the SEM images, could further explain the similar behavior of PUS composites for other mechanical properties.

The Young’s modulus was then tested for neat PU and PUS composites as presented in Figure 4b. Young’s modulus is the measure of elasticity of the material which could reach infinity for a perfectly rigid material. The PUS composite reaches its highest rigidity of 1388.28 MPa or becomes very stiff for starch contents up to 1.5%. On both sides of 1.5 wt.% starch content, PUS composites show less rigidity and better elasticity. The starch fillers were bonded to the polyurethane molecular chain so that the mobility of the molecules was restricted [26]. Therefore, the flexibility is reduced and stiffness is increased.

The maximum load beyond the yield that PU or PUS composites can hold before breaking down is shown in Figure 4c. This type of measurement is related to a certain extent, to the elasticity and brittleness of the sample under test. The PUS-1.5% composite can hold the maximum load of 288.55 N. Other samples, including neat PU, show less maximum load. The results showed that maximum load of PU improved by about 17% which is the same improvement as observed in the tensile strength, as discussed earlier. The results also show that loading up to 2.0 wt.% starch was not enough to bring the PUS composites to the original status as it shoes an improvement of about 15%. The findings here support the results of the tensile strength shown in Figure 4a.

The elongation at break measurements shown in Figure 4d is another avenue to look at the elasticity/brittleness behavior of the neat PU and the PUS composites. The neat PU sample shows 1.05% elongation at break, which suggests that neat PU is not completely rigid, but rather it has a mixture of rigidity and elasticity with dominant rigidity. The rigidity decreases as the starch filler is loaded to PU. At PUS-1.5% composite, the sample can be elongated to 1.39% of its original length before breaking down. The overall loading of starch to PU increases the elongation percentage to 1.71% of the original sample length. These findings have a significant importance for choosing a suitable application for PUS composites in the industry. Instead of focusing on the durability of the composites only; the other applications such as packaging or similar applications could benefit from this behavior. The starch affects the properties of PU by easing the rigidity of PU which results in increasing the elongation.

### 2.4. Flexural Test

**Figure 5:**
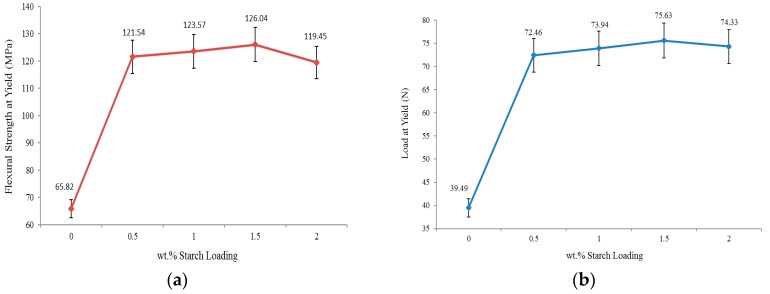

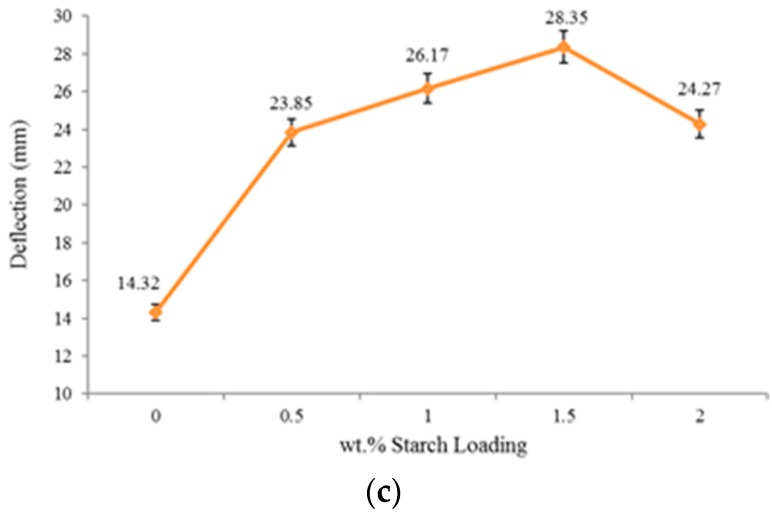
(**a**) Flexural strength at yield; (**b**) load at yield; and (**c**) deflection.

Applying a load in the middle of a sample that is supported at its ends demonstrates a material’s maximum compressive stress and maximum tensile values at the supporting points. The basic requirement of a successful flexural test is that the sample has to withstand maximum tensile strength before failing under compressive strength; otherwise, there is no reason to conduct the flexural test. The flexural test of the neat PU and PUS composites at different starch loading is shown in Figure 5a. The neat PU shows that the flexural test was successfully conducted up to 65.82 MPa compressive stress [27]. The maximum compressive stress that the composites can hold appears at 1.5 wt.% starch, where the stress shows a value of 126.04 MPa. The compressive stress increased by about 91%, and the sample of maximum 2.0 wt.% starch load shows an increase of 81% [28]. This result is in agreement with other results discussed earlier.

The maximum load at the yield of the neat PU and PUS composites is shown in Figure 5b. The maximum yield is defined as the stress where the material begins to deform plastically. Basically, this test is closely related to the elasticity of the material without considering the rigidity. It intends to determine the limits of the performance for mechanical components before partial or total deformation could occur. The maximum load at yield tests help in reshaping the material and explores how susceptible the material is to cutting or shearing. The neat PU sample shows that a load of 39.49 N is enough to deform it, while the PU-1.5 wt.% starch needs 75.63 N to achieve the same level of deformation. Based on this assessment, the PUS at 1.5 wt.% loading shows an increase of 91%, which agrees with the result obtained for the flexural test. The overall results of the maximum load at the yield show clearly that the PU sample is vulnerable to any outside influence such as pressure or loading. The result also shows that even at a loading of 2.0 wt.% of starch, the PUS composite still shows very good elasticity as the yield increases by about 88% compared to the neat PU [29].

Figure 5c shows the deflection caused by a certain load applied on the neat PU and PUS composites. The neat PU can sustain deflection up to only 14.32 mm. As the starch percentage loading to PU increases, the deflection increases by showing better elasticity until 1.5 wt.% at which point the sample shows 28.35 mm deflection. The result here is divergent from other consistent findings of the mechanical measurements discussed earlier, and shown in Figure 4, as 1.0 wt.% starch exhibits the maximum or the critical property instead of 1.5% starch loading. The diversion of the deflection measurement could be attributed to either an experimental error, or more likely, to very similar results between the 1.0 and 1.5 wt.% starch in previous results. The elasticity is improved, based on the neat PU, by about 98%, while the maximum loading of 2.0 wt.% starch shows only 69% improvement. In the shadow of the results here and other mechanical measurements, it is important to add at least one test between 1.5 and 2.0 wt.% starch to a better localize the critical values of the mechanical properties.

### 2.5. Impact Test

The impact test is expressed by the energy or, more specifically, the intensity which is the power per surface area. The basic condition for the impact test is to fracture the specimen by evenly distributing enough load throughout the sample. There are two impact tests; one without a notch and the other with a notch, where the notch is a groove made by very sharp razor with specifications determined by a standard based on the machine itself.

**Figure 6:**
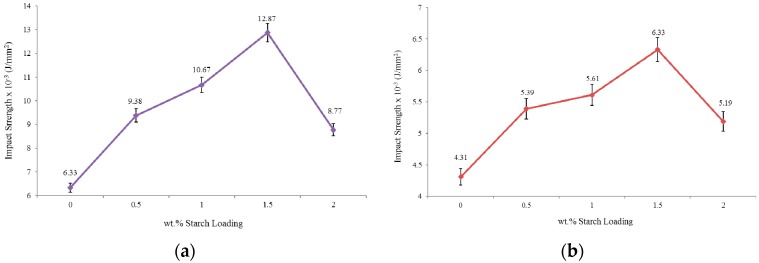
Impact strength (**a**) un-notched specimens and (**b**) single-notched specimens.

The impact strength test of un-notched PU and PUS composites were carried out and the results are shown in Figure 6a. The impact strength of the neat PU is measured at 6.33 × 10^−3^ J/mm^2^ and as starch loading increases, the impact factor increases showing its maximum at 12.87 × 10^−3^ J/mm^2^ at 1.5 wt.% starch loading. The results show an improvement of about 103% compared to the neat PU which, by increasing starch loading, declines to only 38% at the highest starch loading of 2.0 wt.%. Apparently, the morphology of the higher starch loading samples was distorted based on SEM analysis.

The last mechanical test is shown in Figure 6b through which the same impact test was repeated on samples with a notch on the sample surface. As expected, the neat PU sample showed less impact strength at 4.31 × 10^−3^ J/mm^2^, declining by 47% compared to the original un-notched sample. The impact strength increases as starch loading percentage increases for the composites samples and reaches its maximum at 6.33 × 10^−3^ J/mm^2^ and, similarly to un-notched samples, declines for higher starch loading contents.

The effect of notching the sample could be used to show that the smoothness of the sample surfaces plays an important role in the impact strength [30]. The results show that the maximum impact strength which occurs at starch loading of 1.5 wt.%, declines by a significant amount of 103%. This significant decrease is marked in science and engineering as a phenomenon that deserves to be highlighted and studied thoroughly.

For completeness, the results presented in this section are summarized in Table 1 which includes the mechanical tests results for neat PU and PUS at starch contents of 0.5%, 1.0%, 1.5%, and 2.0%.

### 2.6. Dynamic Mechanical Analysis (DMA)

The DMA technique provides viscoelastic measurements of the response of a material under the influence of a sinusoidal oscillation force, which might be in the form of a strain (strain controlled instrument) or a stress (stress controlled instrument). The stress/strain could be in any mechanical form such as tension, compression, or torsion. For a perfectly elastic solid, the strain and the stress are in phase, while in a purely viscous media, the strain lags the stress by 90°. The phase difference lies somewhere between 0° and 90° for all other solid materials depending on the rigidity and elasticity. The storage modulus ($E^{\prime }$) measures the stored energy while the loss modulus ($E^{\prime \prime }$) represents the dissipated energy as heat, for example. The DMA technique is used to identify the mechanical properties of a material in terms of temperature or strain rate (frequency). The region of the linear viscoelastic region (LVR) is used for DMA tests because in this region the modulus is independent of the applied stress or strain magnitude. Generally, the DMA characterizes the glass transition (*T_g_*) temperatures, storage and loss moduli, and heat deflection or softening points.

**Figure 7:**
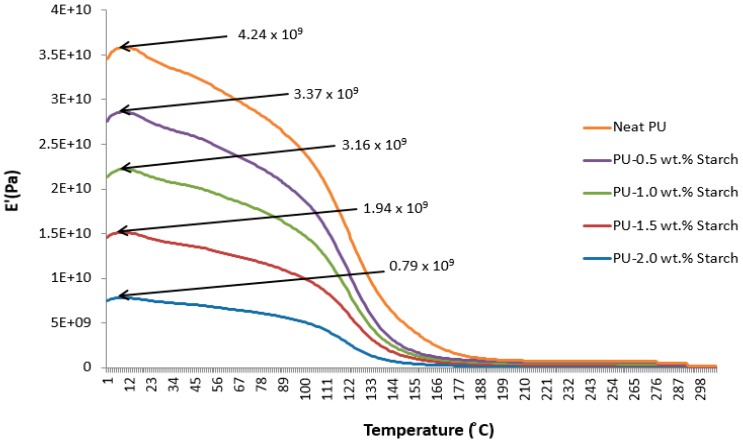
Storage modulus of PU and PUS composites.

Figure 7 shows the storage modulus of neat PU and PUS composites as a function of temperature. All materials show maximum storage ability at about 15 °C, after which the storage modulus is fading out exponentially. The results suggest that the neat PU has the best storage modulus ability which decreases as starch content increases from 0.5 to 2.0 wt.%. The results also show that the highest temperature at which $E^{\prime }$ can be tested for neat PU appears at about 180 °C or lower, while the maximum temperature of PU-2.0 wt.% starch is shifted to a lower temperature of 140 °C.

**Figure 8:**
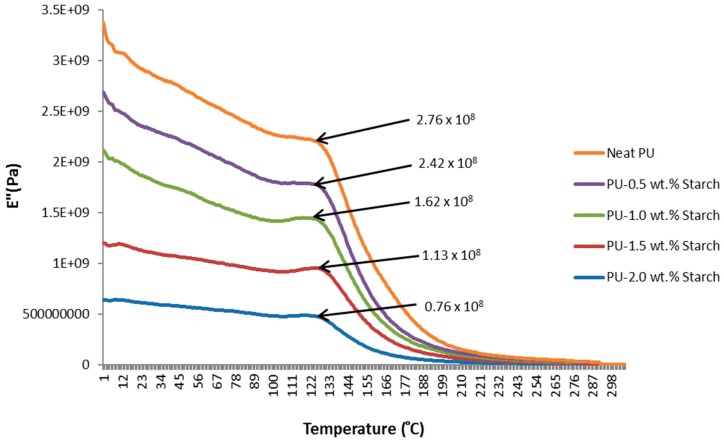
Loss modulus of PU and PUS composites.

The loss modulus ($E^{\prime \prime }$) curves obtained from the DMA instrument for neat PU and PUS composites are shown in Figure 8. The loss modulus curves show that neat PU crystallizes at 124 °C, while PU with the addition of 0.5 wt.% of starch crystallizes at 127 °C. This is indicative that adding a small amount of starch could shift the crystallization temperature (*T_c_*) of PU. Adding starch to a PU matrix enhances the nucleation process of PU crystallization by increasing the surface area of the PUS and providing more nucleation sites for the PU to crystallize.

**Figure 9:**
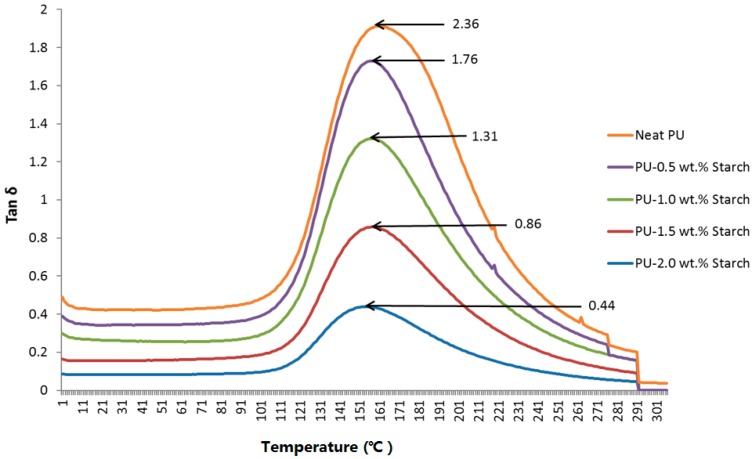
Loss tangent of neat PU and PUS composites.

Figure 9 shows the loss tangent (tanδ), which is defined as the ratio of ($E^{\prime }/E\prime \prime$) of neat PU or PUS composites. The loss tangent is used to measure the melting temperature (*T_m_*). The results show that *T_m_* shifts from 156 to 150 °C for PU-2.0 wt.% starch.

### 2.7. Thermogravimetric Analysis (TGA)

**Figure 10:**
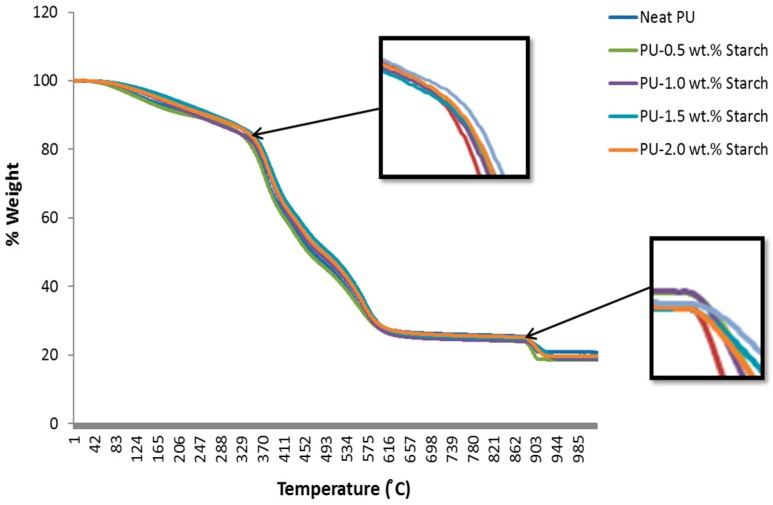
Thermogravimetric analysis of PU and PUS composites.

The TGA curves for neat PU and PUS composites at different loadings are shown in Figure 10. For the PUS, the whole degradation steps can be observed from about 35 to 1000 °C. Generally, the weight loss of the various loadings of PUS composites does not show a significant difference, suggesting that adding small starch quantities to PU has almost no serious influence on the water contained in the matrix. The results show four major regions of the weight loss: between 35 and 360 °C, between 360 and 570 °C, between 570 and 900 °C, and finally between 900 and 1000 °C. The rate of the weight loss in these four regions is: 16%, 52%, 8%, and 5%, respectively. The highest weight-loss rate appears in the second region of 360 and 570 °C. The results also show that minimum weight loss appears in the last region between 900 and 1000 °C. Overall, the PUS composites up to 2.0 wt.% starch do not show a significant difference, which suggests that the elasticity gained for the nanocomposites has almost no effect on the water contained in the matrix.

**Figure 11:**
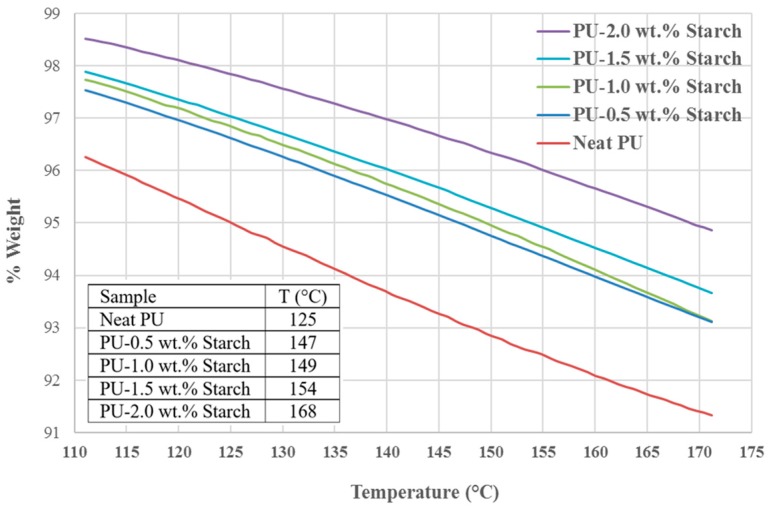
The 5% weight loss of PU and PUS composites at their respective temperatures. The insert shows the temperature at which the sample loses 5% of its weight.

The degradation at $T_{5\%}$ is a scale used to compare the behavior of samples at 5% weight loss due to heating, as pointed out by [31]. Figure 11 and the insert show the temperatures at which PU and PUS composites lose 5% weight. As the starch contents increases, the temperature required to achieve 5% weight-loss increases from 125 °C for neat PU to 168 °C for PU-2 wt.% starch. The increase in temperature reflects the effect of the starch as an additive to PU which makes PU more resistive to increasing temperature or more stable construction.

## 3. Materials and Methods 

There are only two materials included in this paper: thermostat PU and natural starch in powder form. The most important properties of PU and starch are listed in Table 2. Thermostat PU was obtained from Global Innovations-Polycarbonates Bayer Material Science AG, D-51368 Leverkusen, Germany. The starch powder is an organic material; unmodified maize starch (Gelose 80) was purchased from Sigma-Aldrich, St. Louis, MO, USA.

### 3.1. Sample Preparation and Experimental Set-Up

**Figure 12:**
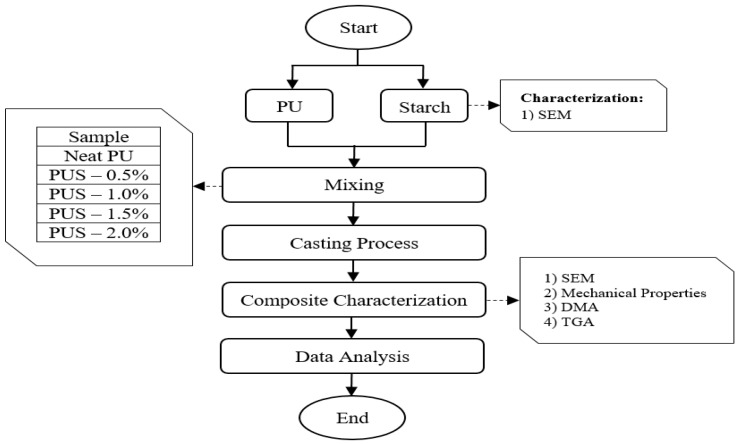
Sample preparation and experimental set-up.

The sample preparation and the experimental procedures were conducted according to the flow chart shown in Figure 12. The imported thermostat PU was taken and mixed at weight percentage with the natural starch at 0.5, 1.0, 1.5, and 2.0-weight percentage. The mixture of PU and starch (PUS) of each sample was manually mixed (casting) and with the aid of heat formation during the mixing process, homogeneous solutions were obtained. In addition to the neat PU sample, four samples were prepared by loading starch with PU at 0.5, 1.0, 1.5 and 2.0 wt.%. These samples (composites) are named as PUS-0.5%, PUS-1.0%, PUS-1.5%, and PUS-2.0%.

### 3.2. Instrumentation

The morphologies of the composites’ fracture surfaces were studied using a scanning electron microscope (SEM), Hitachi TM 3000, Somerset, NJ, USA. The 3-D SEM images are, therefore, useful for judging the surface structure of the sample. Tensile properties were determined using an Instron universal testing machine (INSTRON 5567, a product of Konigsallee, Düsseldorf, Germany), with a 200 Newton load transducer, according to the ASTM D-638 type V method standard [32]. The tensile tests are conducted at a crosshead speed of 50 mm/min and at room temperature. The three-point bending test (flexural test) was carried out using 100 kN Universal Testing Machine according to specifications stated in Instron 8801 (UK). The speed of the test was set at 10 mm/min. The shape of the specimen used for the flexural test was investigated by ASTM D790. The Charpy impact strength tests for both un-notched and single-notched specimens at room temperature were carried out using ASTM D5942 equipped with a pendulum impact machine (MT3016, UK). A pendulum with Terco-15 J was selected for the impact tests. Dynamic mechanical measurements (DMA) (Waltham, MA, USA) were determined using Pyris Diamond DMA which consisted of a temperature programmer and controller. Samples with dimensions of 11 mm × 20 mm × 2.9 mm prepared by compression press were used for analysis. It measures dynamic modulus, both storage (*E′*) and loss modulus (*E′′*). DMA spectra were taken in tensile mode at 1 Hz frequency in a broad temperature range (50 to 150 °C) with a programmed heating rate of 2 °C/min using a Dual cantilever method, the force applied was 6.35 N, displacement 20 μm. Finally, thermal stability and composition were assessed using Thermogravimetric (a product of TA Instruments, New Castle, US) (Perkin-Elmer Pyris 1 TGA, 30–1021 °C at 10 °C/min under nitrogen with the change to air at 700 °C). Samples of about 7–8 mg each were heated from 50 to 950 °C at a rate of 10 °C/min under nitrogen atmosphere.

## 4. Conclusions

Improving the mechanical and thermal properties of PU is very important due to an essential demand for industrial and technological use. The thermo-mechanical properties are improved by adding starch to PU at very small amount of 0.5, 1.0, 1.5, and 2.0 wt.%. The effect of loading starch was tested thermally and mechanically by several techniques, such SEM, tensile strength, elongation, Young modulus, maximum load at yield, and DMA of PU and PUS. SEM images of the fracture surface have shown two features: the formation of agglomerated starch over the surface and the potentiality of PUS composites to having cracks at about 1.5 wt.% starch loading. The mechanical properties were improved by 17% for the tensile strength, and about 91% for maximum load at the yield before being adversely reduced due to the agglomeration and cracks. Even the mechanical properties declined after 1.5 wt.% starch loading, however, the mechanical properties never returned the PUS composites to the standard of PU alone. To show the effect of the natural cracks, a test was performed to investigate the effect of creating notches on the PUS surface. The result showed a significant effect of such an artificial crack by reducing impact strength by 103% compared with a non-notched sample. This result shows the importance of the smoothness of the surface, as well as the prevention of the formation of cracks. DMA analyses of storage and loss modulus have shown that the crystalline temperature and melting temperature were affected. These results may lead to expanding the use of PUS composites.

## Figures and Tables

**Table 1:** Summary of the mechanical tests.

Test		Starch %	0	0.5	1.0	1.5	2.0
	Type of Test						
Tensile	Tensile Stress (MPa)		8.19	8.51	9.35	9.62	9.45
	Young’s Modulus (MPa)		1068.86	1185.31	1310.69	1388.28	1262.08
	Maximum Load (N)		245.69	255.13	280.41	288.55	283.52
	Elongation at Break (%)		1.05	1.16	1.21	1.39	1.71
Flexural	Flexural Stress at Yield (Mpa)		65.82	121.54	123.57	126.04	119.45
	Load at Yield (N)		39.49	72.46	73.94	75.63	74.33
	Deflection (mm)		14.32	23.85	26.17	28.35	24.27
Impact	Impact Strength un-Notched × 10^−3^ (J/mm^2^)		6.33	9.38	10.67	12.87	8.77
	Impact Strength Single-Notched × 10^−3^ (J/mm^2^)		4.31	5.39	5.61	6.33	5.19

**Table 2:** Physical and chemical properties of PU and starch.

Property	PU	Starch
Chemical Formula	C_27_H_36_N_2_O_10_	(C_6_H_10_O_5_)n
Chemical Structure	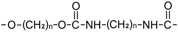	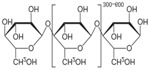
Molar Mass (g/mol)	548.589	Variable
Appearance	Yellow	White
Density (g/cm^3^)	1.2	1.5
